# Supplementary material for: Social Location and Decision-Making Among Women Living with HIV in the Southern United States: An Intersectional Approach
Source: Int J Environ Res Public Health. 2024 Nov 27;21(12):1575. doi: 10.3390/ijerph21121575 (PMC11675124; doi:10.3390/ijerph21121575)
Supplement: Supplementary file 1 [file ijerph-21-01575-s001.zip › ijerph-3229928-supplementary.pdf]

In-depth Interview Guide

Study code number: __________

Pseudonym: _________________

Date/time: ___________________________

Interviewer: __________________________

Question Group 1:

Please tell me how long you have been living with HIV.

Do you have a regular source of healthcare to help you manage your HIV?

> If yes, what is the nature of that source (HIV Clinic, Emergency department, episodic care, primary care, etc.)?

What kinds of things, if any, help (or would help) you to attend your HIV care appointments? (e.g., car, babysitter, time off from work)

What kinds of things, if any, make it more difficult for you to attend your HIV care appointments? (e.g., no transportation, no babysitter, can't leave work)

What kind of things, if any, make it easier (or would make it easier) for you to take your HIV medicine?

What kinds of things, if any, make it more difficult for you to take your HIV medicine?

Does the way you receive your HIV medication refills (pharmacy, mail, etc.) make a difference in how likely you are to take medication every day?

Question Group 2 (geographic location):

In what ways, if any, does where you live (rural/non-rural area) make a difference in your experience of living with HIV?

In what ways, if any, does where you live (rural/non-rural area) make a difference in your ability to care for your HIV (whether you seek treatment)? Attend your healthcare appointments? Take your HIV medicine?

Question Group 3 (socioeconomic status):

In what ways, if any, do you feel your resources, financial or otherwise, make a difference in your experience of living with HIV?

In what ways, if any, do you feel your resources, financial or otherwise, make a difference in your ability to care for your health (whether you seek treatment)? Attend your healthcare appointments? Take your HIV medicine?

Question Group 4 (gender identity):

Please tell me specifically about your experience of being a woman living with HIV.

Have you experienced discrimination based on your gender identity as a woman living with HIV? Have you been treated differently?

Probes:
If reports gender discrimination -
Can you please describe your experience(s)?
In what ways, if any, have such experiences impacted whether you seek treatment for your HIV?
Have such experiences impacted you taking your HIV medication? If so, please describe.
Have such experiences impacted you attending your healthcare appointments? If so, please describe.

Question Group 5 (racial/ethnic identity):

Please tell me specifically about your experience of being a (Black/Latina/White) person living with HIV.

Have you experienced discrimination based on your identity as a (Black/White/Latina) person living with HIV? Have you been treated differently?

Probes:
If reports discrimination/racism based on race/ethnic identity -
Can you please describe your experience(s)?
In what ways, if any, have such experiences impacted whether you seek treatment for your HIV?
Have such experiences impacted you taking your HIV medication? If so, please describe.
Have such experiences impacted you attending your healthcare appointments? If so, please describe.

Question 6 (HIV-related stigma):

Stigma definition: experiences of being discredited, dishonored, or shamed because of who you are or how you identify

Have you told anyone about your HIV diagnosis? If so, who? [not names, just generally who the people are…spouse, child(ren), etc.]

Have you been stigmatized by others for living with HIV?

Probes:
If reports being stigmatized -
Can you please describe your experience?
In what ways, if any, have such experiences impacted whether you seek treatment for your HIV?
Have such experiences impacted you taking your HIV medication? If so, please describe.
Have such experiences impacted you attending your healthcare appointments? If so, please describe

Question 7 (Intersection of Identities):

Is there a combination of these things (being a woman, being Black/White/Latina, living rurally/non-rurally, etc.) that have made a difference in your experience of living with HIV? How you are treated?

Please tell me more about that/those.

Question 8 (Wrap Up):

Is there anything else you would like to tell me today?

Field Notes:

Record information describing the interview experience, including any impressions or events that occurred during the interview.

Length of Interview:

Where did interview take place?

Who was present during interview other than participant?

Describe any interruptions that occurred:

Describe the participant’s emotional and/or physical state during interview:

Describe any reflections or preliminary thoughts that you have about this interview that you feel are relevant to the analysis of this interview (bullet points for quantitative team):
